# Supporting Primary School Children with Juvenile Idiopathic Arthritis: A Qualitative Investigation of Teaching Staff Experiences

**DOI:** 10.3390/children8070555

**Published:** 2021-06-28

**Authors:** Abbie Jordan, Konstantina Vasileiou, Ceri Brown, Line Caes

**Affiliations:** 1 Centre for Pain Research, University of Bath, Bath BA2 7AY, UK; 2 Department of Psychology, University of Bath, Bath BA2 7AY, UK; k.vasileiou@bath.ac.uk; 3 Department of Social Work, University of West Attica, 12241 Athens, Greece; 4 Department of Education, University of Bath, Bath BA2 7AY, UK; c.l.brown@bath.ac.uk; 5 Faculty of Natural Sciences, University of Stirling, Stirling FK9 4LA, UK; line.caes@stir.ac.uk

**Keywords:** juvenile idiopathic arthritis, teachers, support, qualitative, content analysis, school

## Abstract

Background: Juvenile idiopathic arthritis (JIA) has a deleterious impact on numerous areas of children’s lives, including school functioning. This study moves beyond eliciting child reports of school functioning to examine teaching staff’s experiences of supporting a child with JIA in school. Methods: A total of 51 UK-based teaching staff members with experience of supporting a child aged 7–11 years with JIA in school were recruited. Participants completed an online qualitative survey regarding their perceptions and experiences of supporting a child with JIA in school, with a subsample of 9 participants completing a subsequent telephone interview to explore responses in greater detail. Survey and interview data were analyzed using the conventional approach to qualitative content analysis. Results: Analyses generated 4 themes: (1) communicating, (2) flexing and adapting, (3) including, and (4) learning and knowing. Findings highlighted the importance of clear communication between teaching staff and parents in addition to the need for teaching staff to provide individualized support for children with JIA which maximized their inclusion within the class. Conclusions: This paper provides new knowledge regarding how teaching staff adopt proactive and creative strategies to support children with JIA, often in the absence of appropriate training, identifying support needs and resources for teaching staff.

## 1. Introduction

Juvenile idiopathic arthritis (JIA) is the most common childhood rheumatic disease, with incidence rates of 1.6–23 per 100,000 children [1] and 12,000 children and young people living with arthritis in Britain [2]. Recent work has suggested that JIA may be difficult for clinicians to assess, suggesting that it may reflect four different subtypes of rheumatological disorders rather than a single entity [3]. JIA affects numerous areas of young people’s lives, with potentially negative influences on their physical, social, and emotional wellbeing [4]. Due to the fluctuating nature of the pain and fatigue symptoms and associated disability characteristics for JIA [5], there is greater potential for children with JIA to experience disruption to their schooling journey compared with children with other long-term conditions [6].

Receiving appropriate support for those potential disruptions is essential as education is a critical component in children’s development, with children having a fundamental human right to accessing education [7]. More broadly, the school environment plays an influential role in children’s lives through encouraging the development of independence, identity, cognitive–emotional functioning, and social skills [8,9]. Schools are a central place for children to learn how to form friendships and social competency, both of which are fundamental to positive long-term functioning and wellbeing in adulthood. Additionally, school is often the first social context that children will experience outside of the home, and educational inclusion is therefore central to children’s participation in society. Consequently, absence from school and disruptions to engagement with educational opportunities can negatively impact many areas of children’s lives.

Accumulating evidence highlights how children with JIA report significant school absence due to JIA [10,11,12], with school absence associated with more severe disease in subsequent years [9]. In addition to school absence, children also report experiencing challenges with managing JIA in a school setting. These include child reports of difficulties with physical education, pain, fatigue, and other physical challenges such as writing [9,13]. Such impacts are far-reaching in childhood but also extend into later life. For example, research evidence from a large historical longitudinal study of individuals diagnosed with JIA in childhood showed lower educational achievements in patients with JIA compared to individuals in the general population (without JIA). Additionally, unemployment rates were slightly higher for individuals with JIA who experienced a longer disease duration and were still currently engaged in treatment compared with individuals with JIA who no longer received treatment and/or had a shorter disease duration [14].

Existing literature has contributed to developing an understanding of how children and adolescents with JIA experience challenges with school attendance, engagement, and educational attainment. However, most studies have solely elicited the child’s report concerning challenges with school engagement. To date, no studies have examined teaching staff’s perceptions and experiences of supporting a child with JIA in a school setting, which is essential in informing solutions. Looking at the broader pediatric literature, two studies have examined the role of teachers in supporting a pupil with chronic pain in the classroom [15,16]. These American and Irish studies highlight how teachers are aware of their central role in supporting effective pain management in school settings and acknowledge the constraints that prevent them from offering a supportive environment for school children who live with chronic pain. While useful, such studies are not specific to the requirements of supporting a child with JIA in a school setting as JIA management includes specific characteristics which may differ from those of management of chronic pain such as joint flares and management of biological therapies. Additionally, most studies which have looked at the school-related experiences of children and young people with JIA have included age-related eligibility criteria which span both primary and secondary school ages (e.g., [10,11]). Such a wide age range neglects to acknowledge the many differences between primary and secondary schools. This is important as unlike those in secondary school, children in primary schools typically retain the same teacher, class peer group, and classroom for the whole school day across the school year. Thus, teaching staff in primary school settings are key stakeholders and are likely to offer rich experiential knowledge around how to support a child with JIA in school due to spending a significant amount of time with the child who lives with JIA.

Subsequently, there is a dearth of knowledge concerning teaching staff’s experiences of supporting a child living with JIA in a primary school setting. Our study seeks to address this knowledge gap by exploring the experiences of UK-based teaching staff who support a primary-school-aged child with JIA in a school setting, using qualitative survey and interview methods.

## 2. Materials and Methods

### 2.1. Study Design

A multimethod, cross-sectional, qualitative survey and follow-up interview study was designed for the purposes of exploring teachers’ experiences of supporting a young child with JIA in a primary school setting. To collect the data, a qualitative online survey with teaching staff was conducted. Qualitative surveys can be used to answer a range of research questions relating to experiences, practices, understandings, and perceptions [17]. This means of qualitative data collection allows researchers, through open-ended questions, to capture diverse and rich views which are more focused on the topic of interest compared to other qualitative methods of data collection [18]. Follow-up, semistructured interviews were subsequently conducted with a subset of the survey participants with a view to delving deeper into participants’ experiences and reasoning. The research was reviewed and approved by the Research Ethics Committee of the Department of Psychology at University of Bath (code: 19-208).

### 2.2. Participants and Recruitment

UK-based primary school teaching staff for years 3–6 (typically children aged 7–11 years) who had experience of supporting a child with JIA were eligible to take part in the qualitative survey. An opportunity, criterion sampling approach was adopted to recruit participants. The recruitment strategy involved numerous approaches, including through social media (e.g., Twitter), teaching-related organizations, online newsletters, and email contact with UK-based schools. Schools were eligible for inclusion in the study if they were based in the UK and educated pupils aged 7–11 years. Secondary schools (schools with a first intake of pupils at 11 years of age onwards) were excluded from the study. The researcher contacted eligible schools via online lists of schools in local authorities. Email contact was instigated through the email address provided on the school website. The email requested that the study invitation be sent to the Special Educational Needs Coordinator/Special Educational Needs and Disability Coordinator (SENCO/SENDCO) or any teachers supporting a child with JIA. All study promotional materials included basic information about the research, a link to the survey (including QR code), and the study-specific email to invite any questions about taking part. Consequently, all interested participants were able to directly access the survey from the initial recruitment material if they wished to do so.

In total, 55 participants completed the survey between September 2019 and March 2020. All participants had taught primary school children (4–11 years); ten had also had experience of teaching secondary school children (11–18 years), and one volunteer had also taught sixth form/college students (16–18 years). Of the 11 participants who had also taught in secondary education or sixth form/college students, 4 indicated that the child with JIA whom they had supported was in secondary school; therefore, these participants were excluded from the analysis, leaving 51 eligible respondents. Sample sizes for qualitative surveys vary and are dependent on a multitude of factors comprising the topic, research question, diversity of the population, and detail of participant responses. Given the desire for detailed responses, our sample size was congruent with acceptable sample sizes for qualitative surveys, which typically range between 29 and 100 responses [18].

Table 1 presents the demographic and job-related information provided by the final sample of 51 teaching staff, of whom 43 (84.3%) were women. The majority of participants (*n* = 42; 82.4%) had supported one child with JIA in the school setting; six participants (11.8%) had had experience of supporting two children with JIA, and only two teaching staff (3.9%) mentioned that they had taught three different children with JIA.

A subset of nine survey participants, who in the survey stage had indicated their interest in being contacted for a follow-up interview, was purposively selected and took part in qualitative semistructured interviews between March and June 2020. This subsample of participants was selected to reflect a diversity of teaching roles and experiences from the interested participants who were available within the timeframe of the data collection process.

Eight interviewees were women, one was a man, and all self-identified as Caucasian and were married, except for one who indicated single/never married. Seven interviewees were teachers, and two held teaching assistant positions. The average interviewees’ teaching experience was 17.2 years (SD = 7.5; min = 6 years; max = 26 years). Of the nine interview participants, seven had taught one child with JIA, and the remaining two had each managed two cases of children with JIA in a primary school setting.

### 2.3. Data Collection

The qualitative survey was administered online using Qualtrics, online survey software [19]. The first part of the study provided information about the study and consent statements. After the participants had read the information and endorsed the relevant screening questions and consent statements, they could access the survey questions. All participants had the right to withdraw up to the point of submission of the survey without consequence or need for explanation. As completion was anonymous, once the survey had been submitted, withdrawal was no longer possible. The survey included three domains of questions (see Appendix A: survey questionnaire): the first domain collected demographic (i.e., gender, age) and work-related information (e.g., job title, work role, years of experience) using both close and open-ended questions (*n* = 9). The second domain included questions (*n* = 11) relating to participants’ experiences in supporting a child with JIA at school. The last set of the survey questions (*n* = 9) concerned teaching staff’s support and training needs in relation to JIA. All participants were offered the chance to enter a prize draw with a prize of a GBP 50 shopping voucher once they had completed the survey. Survey content was directly informed by pilot discussions with a small number of teachers to ensure that content was appropriate.

For the qualitative interviews, a semistructured interview protocol was developed to guide the conversations (see Appendix B for the full interview schedule). Participants were provided with the opportunity to ask the interviewer any questions they had and were asked to confirm their consent to take part in the interview. Interview questions addressed the following areas: participants’ experiences of supporting a child with JIA at school; the impact of JIA on children’s education, learning and academic engagement, and strategies teaching staff used to assist them; and the impact of JIA on children’s social and emotional wellbeing. Qualitative interviews were conducted over the telephone to facilitate inclusion of participants across the UK. Telephone interviews have been demonstrated to be a methodologically robust method for generating qualitative data [20]. Interviews were audio-recorded and transcribed verbatim. Interview duration ranged between 22.45 and 41.13 min, with a mean interview duration of 30.61 min (SD = 7.12 min). All participants were provided with a debrief sheet at the end of the interview. Basic demographic information was collected through a short form that participants completed and emailed to the researcher before the interview. Interview participants were offered a GBP 10 shopping voucher as a token of appreciation for contributing to our research.

Both the survey questionnaire and the interview protocol were designed in consultation with the relevant literature and JIA-specific charities. This ensured that questions addressed relevant topics.

### 2.4. Data Analysis

Qualitative content analysis was used to process both the interview and survey data, which we treated as one corpus of data [21]. Qualitative content analysis is a well-established and widely used technique for interpreting meaning from textual qualitative data [22] and is characterized by three distinct approaches: conventional, directed, and summative [21]. In this study, the conventional approach to qualitative content analysis was adopted [21], whereby the codes are generated inductively from the data. The conventional approach to qualitative content analysis is a suitable analytic choice when the research seeks to describe the phenomenon of interest while staying grounded in the actual data [21].

The initial familiarization process with the data was conducted by KV through repeated reading, and the open-ended data from the survey were coded manually using a typical word processing package. This initial coding scheme was then revised twice, with codes reviewed and refined on each occasion to best reflect patterns in the data. Between these processes, codes were discussed with AJ. The interview data were processed and coded with the assistance of NVIVO 12 software [23]. Initial codes from interviews were reviewed and refined ensuring that the final coding scheme reflected the data of interest. Having completed the coding of all data, the researchers then produced a preliminary analytic report that grouped related codes into broader categories related to our research questions, that is, the support strategies that teaching staff use to help a child with JIA and the teachers’ training needs. This preliminary analysis was then discussed among the authors, revised, and refined with a view to satisfying the criteria of internal homogeneity and external heterogeneity [24] of the developing categories. The final results are presented in the next section with illustrative extracts cited to support our analytic insights. Extracts are identified using the participants’ unique code and indicating whether they are sourced from the survey or the interview data. The iterative nature of the analytic procedures and discussion of findings with authors ensured that the analyses were both grounded in the data and interpretations were credible, providing evidence of the quality of the analyses [17,25].

The demographic and job-related data collected from the closed questions in the survey were inserted into IBM SPSS for Windows Version 27 [26], and descriptive statistics were computed (i.e., frequencies, percentages, means, standard deviations).

## 3. Results

**Figure 1:**
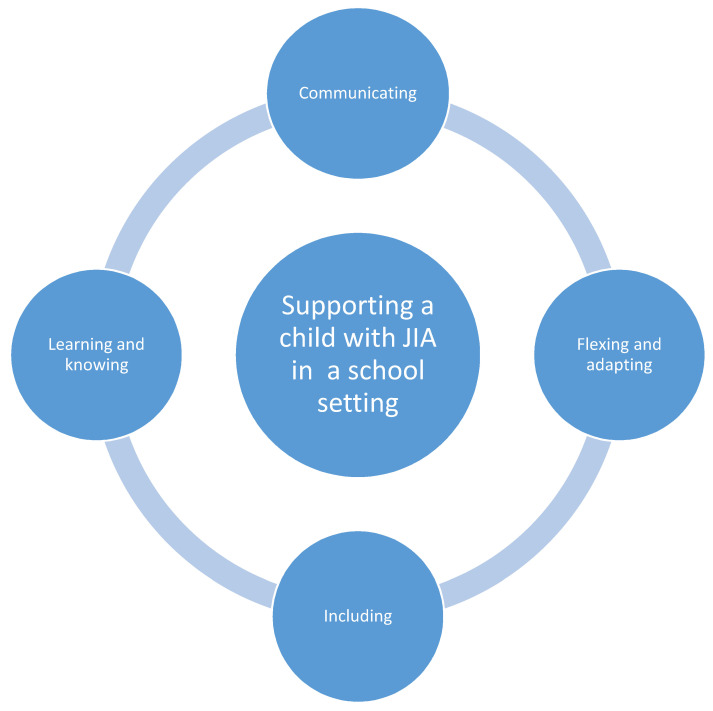
Visual representation of themes representing teaching staff’s experiences of supporting a child with JIA in a school setting.

Qualitative content analysis of survey and interview data generated four themes which represented the experiences of teaching staff regarding supporting a child with JIA in a primary school setting. These four themes comprise (1) communicating, (2) flexing and adapting, (3) including, and (4) learning and knowing. Each theme is subsequently described in detail below, with extracts from survey responses and interview data provided as exemplars of findings. Figure 1 provides a visual overview of the four themes.

### 3.1. Communicating

Clear communication with a range of stakeholders was critical in terms of the ability of teaching staff to support a child with JIA in a primary school setting. Stakeholders comprised children with JIA, parents, fellow teaching staff, healthcare professionals, and school pupils. Good-quality communication was especially important since teaching staff reported a lack or at best minimal knowledge of JIA and how to support a child with JIA in school. Participants described parents as the most important source of information, with regular parent–staff communication enabling individuals to understand the child’s unique, individual needs and to collaboratively design strategies to support the child in school.

*“All the support put in place has been through discussions with the parents and ideas that they have that can support her. We are in constant communication”.*

(P45—survey)

The emphasis on good communication patterns between teaching staff and parents underscores teaching staff’s awareness of the importance of acknowledging the unique presentation of JIA and its impact on the individual child.

*“The most important thing was to talk to the child and parents about the specific issues they faced. It seemed that each sufferer was very individual”.*

(P22—survey)

Acknowledging the individualistic impact of JIA on children highlighted how teaching staff positioned parents as the “experts” regarding how best to support the child with JIA. To accommodate efficient sharing of information between parents and teaching staff, communication occurred at different levels. Daily communication about the child’s functioning enabled teaching staff to tailor practices according to the child’s specific abilities on a particular day.

*“Mum and myself communicated on a daily basis so I was always aware exactly how she [child with JIA] was feeling before commencing the school day”.*

(P06—survey)

Contrastingly, communication also occurred at a broader level, focusing on collaborative discussion between parents and teaching staff around global strategies such as use of aids to support the child. The importance of clear parent–teaching staff communication was particularly crucial in situations where teaching staff did not receive JIA-specific information from healthcare professionals. In such instances, parents adopted a mediating role between both parties to support the child with JIA within a school setting.

*“After diagnosis, I was able to research and was given some information from the paediatrician via parents”.*

(P09—survey)

Beyond the role of parents, fellow teaching staff fulfilled a critical role in maximizing communication around supporting a child with JIA in school. In particular, previous teachers shared the condition-specific knowledge they gained through their personal experience of supporting the pupil and how this information may be used to support the child within school.

*“Initial communication with the previous teacher with regard to how and when to differentiate, the impact of his condition in the classroom and any strategies they may have previously used”.*

(P15—survey)

Communication between teaching staff and the child with JIA was also critical, particularly regarding managing the child’s pain. Teaching staff worked collaboratively with children to identify suitable strategies to enable them to recognize when the child was experiencing pain. Strategies included use of a faces pain scale, or the traffic light system described below. Effective communication of these strategies enabled teaching staff to take appropriate action to address the child’s pain (e.g., change of activity). Further detail of such strategies can be found in Appendix C.

*“With the traffic light system, red was sore, orange was okay, and green was really good. It was very simple for her to be able to say, ‘I’m green, I feel really okay today. I’m not too good, so it’s the orange. Red is, I’m feeling really sore”.*

(P07—interview)

Clear communication between teaching staff and children with JIA focused on teaching staff actively listening to the child regarding the unique challenges that living with JIA placed on their engagement with school. This enhanced sense of understanding (teaching staff) and being understood (children with JIA) enabled teaching staff to consider how they could most appropriately talk about JIA with pupils to enable classmates to support their peer.

*“We also spoke to the pupils in the class about his arthritis so that they had an understanding and could consider it when they were working with him”.*

(P37—survey)

In conclusion, this theme highlights the importance of clear communication between teaching staff and all stakeholders, identifying the key role that parents play in providing information to enable teaching staff to best support children with JIA within a school setting.

### 3.2. Flexing and Adapting

In addition to effective communication, another key aspect of supporting a child with JIA in a primary school setting focused on adjusting learning practices to accommodate for the specific challenges of managing JIA with a particular child. Such adaptations varied in nature, ranging from adjusting physical activity sessions to classroom arrangements and extracurricular activities. In relation to physical education (PE) classes, some participants adapted the curriculum, noting how they “*differentiate the task in PE to reduce impact on the joints” (P47—survey).* At a broader level, teaching staff adopted a flexible approach in devising and implementing support strategies, enabling the child to participate in school life to the best of their ability. Strategies included flexibility around “*adapting the timetable or curriculum” (P22—survey)* where required to meet the child’s needs. Additionally, participants described the need to implement adaptations at a more individual level to enable the child to engage to the best of their ability, describing how they take “*the lead from how the child was feeling on that particular day” (P50—survey).* An example of this is splitting academic work into smaller tasks when concentration levels were adversely affected or giving extra time to complete the task. This required teaching staff to be alert to the child’s current physical and psychological wellbeing and to adopt a proactive attitude to supporting the child.

*“He [child with JIA] wouldn’t put his full effort into certain activities, which wouldn’t be like him because he was always one for trying really hard. I would then have a wee conversation. ‘Are you feeling a bit tired today?’ ‘Yes.’ ‘That’s fine. Just do what you can and leave what you can’t do.’ It is having a little understanding and awareness”.*

(P18—interview)

Dependent on the presentation of JIA, a particular challenge for children with JIA related to writing. Teaching staff were required to be vigilant, looking for instances where the child appeared to be struggling. For example, children’s use of handwriting aids such as *“chunkier pencils (P08-interview)* or pencil grips, often indicated to participants that children were feeling uncomfortable. In such instances, teaching staff offered additional strategies such as “w*riting breaks if needed and additional time to complete tasks” (P15—survey)* or use of an iPad or a scribe to reduce the burden on the child with JIA.

*“My TA [teaching assistant] or I can scribe if we are writing a larger piece of text—or they will type”.*

(P26—survey)

An integral element of teachers’ flexible approach to supporting children with JIA was the practice of empowering children to maximize their own engagement in school life by providing them with a range of support “options”. For example, sitting was often a problematic area for children with JIA and a key activity within the school day. To address this, participants provided children with multiple adaptive options, including sitting on a chair, a bench, or a cushion instead of sitting on the floor cross-legged or standing up during the assembly. One participant described how they ensured that the child “*had a seat to sit in rather than sitting on the floor, if they wanted to” (P09—interview)*.

While many adaptations focused on regular school activities such as writing and sitting, teaching staff were also required to adopt a flexible approach to managing less frequent situations such as school trips. In such instances, participants were required to plan ahead to maximize inclusion of the child with JIA, requiring them to consider issues such as the amount of walking involved. Adaptations were often discussed with parents, with some parents adopting an active role in the mutually agreed adaptive strategies to maximize the child’s engagement.

*“Her mum always came on school trips for extra support and with her chair so she could use that when walking became too much”.*

(P06—survey)

To conclude, this theme highlights the importance of teaching staff adopting a flexible and creative approach to supporting a child with JIA, recognizing the importance of meeting individual needs to enable children to succeed and engage in school settings.

### 3.3. Including

Teachers described tension between supporting the child’s inclusion in school and remaining mindful of the need to ensure that the child was not “marked out as different” from peers. Hence, teaching staff were cautious while implementing support strategies so as not to draw attention to the child with JIA. For example, P07 describes below how making use of an additional member of staff in the classroom managed this tension.

*“If anything was wrong, there was an extra member of support staff there that predominantly was for her. However, we could share that support with the children that have got needs within the classroom, and that has certainly really worked. We didn’t want her to stand out”.*

(P07—interview)

Minimizing the use of strategies that highlighted the child’s difference from peers was perceived by teachers to be critical since such a situation might result in the child refusing available support. In line with English Special Educational Needs and Disability Policy on participation (see Sections 1.31 and 1.34) [27], some teaching staff attempted to foster inclusion, resulting in changes for the entire class to ensure that the child with JIA was able to engage equally with the activities. For example, P01 describes how a sporting activity was changed to ensure that all children could take part.

*“We took out netballs and everybody had a go so it wasn’t just you’re allowed to use this ball and everyone else is allowed to use this one. So, trying to think of ways of using different equipment but it being part of the lesson”.*

(P01—interview)

However, it was not always possible to implement changes at a class level. In some instances, individual adaptions necessitated physical separation of the child from the peers (e.g., staying inside during lunch break). To mitigate this, teaching staff ensured that, when implementing a strategy that necessitated separation, the child was permitted to select a friend to reduce isolation and difference from peers.

*“She [child with JIA] could have friends go with her to different places so when she was allowed out early from a lesson, she could take a friend with her, so she wasn’t alone”.*

(P02—interview)

Other staff members circumvented this separation by creating additional opportunities to facilitate inclusion when the child with JIA was unable to engage with class-based activities, such as outside-based playing due to cold weather which can exacerbate pain. Highlighting the creative approach adopted by staff to maximize inclusion of the child with JIA, P01 describes an effective social adjustment strategy.

*“I set up a reading group so that she had a social element that was inside, so she could still have a chance to talk to the girls”.*

(P01—interview)

While teaching staff were aware of the need to balance inclusivity and differentiation, for some children, the perception of being considered to differ from peers was substantial. Occasionally, despite efforts by the teacher to provide inclusive support, the child’s perception of difference from their peers resulted in some children not engaging with inclusive strategies, which could in turn negatively impact their health.

*“In many ways they [strategies] worked as they allowed him to be included and ensured he wasn’t missing out on any key learning experiences. However, this pupil just wanted to be like everybody else. He didn’t want to be treated differently or do different things from the rest of the class. He never used the time out card, hated sitting on a chair when everyone else was on the floor and didn’t want to use different materials to other pupils…The resources and strategies were in place but more often than not he chose not to use it”.*

(P37—survey)

This theme has identified the importance of teaching staff adopting an inclusive approach to supporting children with JIA within a school setting, with a focus on minimizing the child’s difference compared with peers. An important element of this focus on inclusivity was participants’ recognition that the need to balance adjustment and differentiation for the child with JIA was uniquely dependent on the physical, emotional, and social needs of the child.

### 3.4. Learning and Knowing

While cognizant of their limited knowledge about JIA, participants adopted an active role in learning about how to best support a child with JIA within school. Many participants searched for relevant information from relevant organizations (e.g., JIA charities), with some relying on their personal or familial experience of rheumatological conditions.

*“I’d never heard of it (JIA) until I taught the first child. My father suffers dreadfully from arthritis, so I often thought of him when working with the children”.*

(P36—survey)

While adult-focused rheumatological knowledge may be useful, its applicability to understanding the impact of JIA on children is limited due to development and condition-specific differences. Such findings identify a clear knowledge gap for teachers pertaining to JIA, subsequently highlighting the importance of meeting this knowledge gap through training and resources. Participants identified multiple knowledge gaps, ranging from broader concerns regarding *“What JIA is. How it affects a child” (P19—survey)* to more specific aspects of JIA management, such as medical aspects of JIA, including awareness of JIA symptoms and signs of a flare-up. Moreover, participants indicated training needs in relation to *“pain management, helping them (children) cope” (P23—survey)* in addition to advice on administering medication and its side effects.

Medical support provided by healthcare professionals in school settings (e.g., school nurse or occupational therapist) provided valuable learning opportunities for teaching staff, enabling them to develop activities that could be incorporated in the school day to support the child with JIA.

*“We also had a physiotherapist visit school and did daily exercises in school as recommended by the physiotherapist”.*

(P39—survey)

Despite noting a lack of training regarding how to address the medical needs of a child with JIA, participants described how they were already implementing strategies to support the child to manage the medical aspects of their condition. For example, participants described numerous ways in which they supported the child in managing their medication. These included administering medication in school, ensuring that pain relief medication was administered at regular intervals, and importantly, the social and emotional management of medication.

*“Supporting parents when the child is reluctant to have routine injections and prepare the child for when we know they are due”.*

(P25—survey)

Teaching staff were aware of the social and emotional impacts of JIA in a school setting, describing a desire to learn more concerning how to support “*a child with a medical need in terms of emotional wellbeing” (P19—survey).* Participants wished to learn how JIA might affect a child’s self-esteem, wellbeing, and mental health. This desire to learn how to best support the child with JIA extended to the wider family, with participants recognizing the critical relationship between teaching staff and parents, through a desire to receive information to enable them to implement *“strategies to support both the child and parents” (P03—survey).*

In addition to emotional and physical domains, participants wished to develop their knowledge regarding how to best support the child with JIA academically. Specifically, participants wanted to know more about the effects of JIA on attention, children’s ability to concentrate, as well as school attendance and showed interest in potential ways to support a “*child when they cannot attend school” (P20—survey).*

These knowledge gaps are detailed in Table 2 and highlight the need for training to enable teaching staff to support a child with JIA to the best of their ability. Staff were creative in suggesting different formats through which the JIA training could be delivered or accessed, emphasizing the importance of identifying the relevant training modality that best met the participant’s needs (e.g., online, printed, in-person). The extensiveness of the material was also a consideration for teaching staff, acknowledging the need to balance sufficient detail to be helpful and the time constraints of teaching. Acknowledging the collective nature of expertise about JIA and its impact on children, participants expressed the desire that information provided be derived from a range of key stakeholders rather than a single stakeholder group. P46 described a desire for “*NHS—information. Support from parent groups, those diagnosed to share experiences etc.*” *(P46—survey).*

To conclude, this theme highlights how teaching staff seek to meet gaps in their JIA-related knowledge and the need for additional credible and accessible resources to enable teaching staff to support children with JIA in a school setting to the best of their ability.

## 4. Discussion

### 4.1. Main Discussion Points

The aim of this multimethod study was to gain an insight into the experiences of UK-based teaching staff who support children with JIA in a primary school setting. Qualitative analyses of the data identified how teachers’ support centralized around (1) good communication with all stakeholders involved, primarily parents; (2) being flexible and adaptive; (3) being as inclusive as possible; and (4) ongoing learning about JIA. While to our knowledge, this is the first study exploring teaching staff’s experiences specifically toward supporting a child with JIA, our findings align with research on teaching staff’s experiences in supporting a child with chronic pain. In particular, our findings further highlight how teaching staff are aware of the (1) role that biological, psychological, and social factors play in understanding a child’s experience of symptoms; (2) critical role of establishing a cooperative relationship, characterized by effective communication, between parents and school staff; and (3) need for official training to understand a child’s symptoms and how they can provide evidence-based symptom management [15,16,28]. Taken together, our findings stress the need and relevance of appropriate and JIA-specific training for teaching staff to ensure teaching staff can offer the support that a child with JIA requires to fully engage with and benefit from their school environment despite the challenges that JIA might pose.

Despite teaching staff identifying many gaps in their understanding of JIA and the need for more formal education on JIA, the staff were very skilled and creative in developing and implementing effective strategies to support children with JIA in the school environment. In the absence of any formal training, teaching staff were able to identify the child’s specific needs and implement support individualized to the child’s needs, with a focus on being flexible and inclusive. A critical component to this post hoc, but individualized, support provision was frequent, clear, and bidirectional communication between parents and teaching staff. Indeed, teaching staff received most of their topical knowledge about JIA and more specific information regarding how JIA impacts the child in question from parents. Such a focus on acknowledging the importance of parents is congruent with recent English Special Educational Needs and Disability policy which has granted greater authority to parents as expert decision makers in their children’s needs [27]. However, study findings identified a clear lack of engagement with the child’s multidisciplinary treatment team. This could potentially be problematic as it infers that the information teaching staff rely on is second-hand and hence has potentially lost accuracy through multiple reports [29]. This indirect information transfer might be particularly problematic for children who grow up within a less supportive home environment. While it is crucial for teaching staff to trust in parents for a comprehensive understanding of the unique experience of their child, direct communication or knowledge transfer from healthcare professionals is crucial to ensure appropriate understanding of JIA and its treatment in general terms. In addition, the availability of direct links with the healthcare team would allow a route for teaching staff to address any concerns they do not feel comfortable sharing with the parents (e.g., concerns about parental responses or advice for teaching staff struggling to provide the support parents request).

Consequently, as identified by the teaching staff in our study, there is a critical need to provide teaching staff with evidence-based, credible information on JIA, multidisciplinary treatment of JIA, the (long-term) impact of JIA on children, and how to support children within a school setting. Ideally, such training should be integrated in primary school teachers’ educational curriculum, but for various reasons, such integration is likely not feasible. Curriculums are notably already dense, JIA is only one of many pediatric long-term conditions that teaching staff might encounter and need to provide support for, and, although JIA is common among childhood rheumatic conditions, most teaching staff might never need to support a child with JIA. Consequently, more realistic approaches would be to offer such knowledge to teaching staff who anticipate the need to support a child with JIA through evidence-based leaflets and workshops, with the due workload relief necessary to undertake training. Crucial in the design of such educational materials will be the co-development with researchers, clinicians, teaching staff, children with JIA, and parents of a child with JIA to ensure the information is not only credible but also reflects the perspective from all relevant stakeholders [30]. Furthermore, providing examples of the creative approaches that current teachers have implemented can offer inspiration to teachers being new to and feeling insecure in this supportive role. While communication was reported to be frequently informal or ad hoc, teaching staff could usefully compile a personalized log of resources and key information that could both act as an aid for the individual child following school transition, as well as for new staff in the case of teacher replacements.

An important part in providing training and support for teaching staff will be on how to address the balance between providing flexible adaptations to support the child with JIA, while being mindful to be inclusive and ensure the child does not stand out or is made to feel different. Indeed, evidence across pediatric long-term conditions, including JIA, highlights how being different from their peers is one of the most difficult things to deal with for children and hence something that they try to reduce at all costs [31]. However, as highlighted by the responses shared by the teaching staff in the current study, providing flexible support that is also inclusive is an extremely difficult balance to successfully achieve. A continuous evaluation of the support strategy is required as children’s symptoms and the impact on their ability to engage with the school activities change frequently and are unpredictable. The reports from the teaching staff involved in this study show how inclusivity was achieved through either involving the whole class in symptom management activities (e.g., the whole class can be engaged in relaxation exercises on several occasions throughout the day) or involving part of the class in alternative activities (e.g., inside reading group). Such inclusive and flexible support also requires an individualized approach attuned to the needs of the particular child and might be a key aspect where teaching staff can learn from each other regarding how they deal with specific situations successfully. Although future research on its effectiveness within this particular setting is needed, guided by the peer support literature [32,33], matching teaching staff who are currently providing support for a child with JIA to teaching staff who have past experience of providing such support could prove worthwhile to improve teaching staff’s confidence and perceived abilities.

### 4.2. Limitations

It is important to consider our findings in the light of several limitations. First, we acknowledge that the study required teaching staff to report on working with children who self-report a diagnosis of JIA. Consequently, there were no means to verify receipt of a formal diagnosis of JIA and/or disease severity of children supported by teaching staff. Second, the generalization of the findings is limited due to a focus on UK-based teaching staff only. Further studies in other countries are needed to explore the similarities and differences in how teaching staff can be supported to provide optimal care for a child with JIA within a primary school setting. Being aware of potential local or country-specific needs is of importance in developing educational materials to support teaching staff. The study focused on teaching staff’s perspective in supporting a child with JIA, but to gain a comprehensive understanding of how to effectively support a child with JIA within a school setting, it would be necessary to explore perspectives of all relevant stakeholders, including parents and children. Consequently, future research focusing on the perspective of children themselves and their parents will be crucial.

### 4.3. Implications

Taken together, these findings add to the growing evidence highlighting the need for appropriate training and support to school staff. Effectively preparing teaching staff to provide support for children with JIA is of relevance as evidence highlights how a positive teacher–pupil relationship, in which students feel their autonomy and competence is respected, supported, and valued by their teachers [34], can have far-reaching effects on children’s academic (e.g., school attendance, functioning, and satisfaction) and social–emotional functioning (e.g., reducing bullying and school-related stress) [34,35]. Consequently, there is an urgent need for evaluations of the implementation and effectiveness of educational materials, co-designed with teaching staff, clinicians, children, and parents, to effectively prepare school staff in supporting a child with JIA in the school setting and thereby preventing a negative impact on their school engagement and functioning in society.

## 5. Conclusions

This study provides novel evidence to highlight the important role that teaching staff play in supporting a primary-school-aged child with JIA in a school setting. Findings highlight the importance of clear communication between teachers and other parties and training gaps regarding maximizing teaching staff’s ability to support a child with JIA within the classroom.

## Figures and Tables

**Table 1:** Survey participants’ demographic and work-related characteristics (*n* = 51).

Gender	Women	43 (84.3%)
	Men	8 (15.7%)
Age (in years)		Mean (SD) = 41.6 (10.3)
		Min = 22; Max = 60
Current job title	(a) Teacher (various levels/seniorities)	36 (70.5%)
	(b) Special Educational Needs Coordinator SENCO	5 (9.8%)
	(c) Teaching assistant positions (various levels/seniorities)	7 (13.7%)
	(d) Primary physical education (PE] specialist	1 (1.9%)
	(e) Other roles (e.g., administration manager)	2 (3.9%)
Years of experience in the current work role		Mean (SD) = 11.5 (8.1) Min = 1; Max = 34
Level of confidence with supporting a child with JIA (1 = totally unconfident; 10 = very confident)		Mean (SD) = 6.9 (1.8)

**Table 2:** Gaps in teaching-staff-reported knowledge concerning supporting a child with JIA in a school setting.

Knowledge Gap Domain	Specific Identified Training Need
Medical and physical	Presentation of JIA and how this differs to other types of arthritis
	How to support the child to manage pain flares
	Short and long-term effects of JIA on the child
	Co-occurrence of JIA with other conditions
	Pain management (how to administer, side effects of medications, effectiveness of non-pharmacological methods of pain management)
	How to support children to use aids in a school setting (e.g., handwriting aids)
Emotional	Potential effects of JIA on a child’s self-esteem and self-image
	How JIA may impact a child’s mental health
	Strategies to support familial management of JIA
Peer relationships	Supporting children’s friendships during breaktimes/lunchtimes
	Educating the child’s peers about JIA
	How to create an inclusive environment among the child’s classmates
Academic	Impact of JIA on children’s attention and concentration
	Supporting school attendance
	How to support a child academically while absent from school

## Data Availability

Data are not available as ethical permission was not obtained from participants for the sharing of their data.

## Appendix A. Survey Questionnaire

**Section A: Participant Demographic and Job-Related Information**	
Question	Type of question (and options of answers where relevant)
(1) Which gender do you identify with?	Closed (1) Male (2) Female (3) Other (4) Prefer not to disclose
(2) What is your age? (age in years)	Open-ended
(3) What is your job title	Open-ended
(4) Can you please briefly describe your work role? (For example, leadership roles, curriculum areas of leadership, SENCO, etc.)	Open-ended
(5) How many years of experience do you have in the above-mentioned role? (Number in years)	Open-ended
(6) Which age groups have you taught/supported throughout your working experience? Please tick the boxes relevant to the age group of children you’ve taught/ supported, including the ones you are currently teaching/supporting.	Closed Primary school Teaching Please note that Scottish school years are placed in brackets (e.g., P5). (1) P1 (2) Y1 (P2) (3) Y2 (P3) (4) Y3 (P4) (5) Y4 (P5) (6) Y5 (P6) (7) Y6 (P7) Secondary school teaching Please note that Scottish school years are placed in brackets (e.g., P5). (1) Y7 (1ST) (2) Y8 (2ND) (3) Y9 (3RD) (4) Y10 (4TH) (5) Y11 (5TH) (6) 6TH (Scotland) Sixth form/College (1) Y12 (2) Y13
(7) What year did you complete your teacher training?	Number indicating the year
(8) What type of training did you complete? Please choose all options that apply:	Closed 1. Postgraduate Certificate in Education 2. Postgraduate Teacher Training Course 3. Professional Graduate Diploma in Education 4. Undergraduate Teaching Degree 5. Future Teaching Scholars 6. Graduate Teaching Programme 7. Now Teach 8. Premier Pathways 9. Researchers in Schools (RiS) 10. Teach First 11. Transition to Teach 12. Other (please specify):
(9) Apart from your teacher training programme, can you briefly tell us about any other training you have completed with regards teaching primary school aged children? For example, have you received training in supporting children with additional needs?	Open-ended
**Section B: Participants’ Experiences in Supporting a Child with JIA in School**	
Question	Type of question (and options of answers where relevant)
(1) Can you please indicate the number and age group of children with JIA you’ve taught so far? Please include the count of the children you are currently associated with.	(1a) Number of children taught with JIA (1b) Age group of children with JIA taught, so far (please tick for current children too): Please note that Scottish school years are placed in brackets (e.g., P5). Q30 (1) P1 (Scotland) (2) Y1 (P2) (3) Y2 (P3) (4) Y3 (P4) (5) Y4 (P5) (6) Y5 (P6) (7) Y6 (P7) (8) Y7 (1ST) (9) Y8 (2ND) (10) Y9 (3RD) (11) Y10 (4TH) (12) Y11 (5TH) (13) Y12 (6TH) (14) Y13
Please answer the questions below in relation to your knowledge of the child or children with JIA that you have supported in a school setting. We realise that this may not be current experience, it’s fine to tell us about previous experience you may have had in supporting a child with JIA in school. Please be careful not to mention any actual names, you can use initials if this makes things easier for you. (2) Can you describe how you came to know of the child’s/children’s diagnosis of JIA?	Open-ended
(3) In your opinion, does having JIA influence a child’s ability to attend, concentrate and engage with school-related activities, such as the following: 1. Academic performance, including the completion of homework? 2. Emotional wellbeing? 3. Playtime and socializing? 4. Movement around the school? 5. Taking part in PE or other sport-related activities? 6. Attending school trips and other activities? 7. Impact of time off due to medical appointments? 8. Other? If so, please specify.	Open-ended
(4) If any, which strategies did you put in place to support the child/children with JIA in your school?	Open-ended
(5) Can you share how useful you believe these support strategies were?	Open-ended
(6) Where there any challenges in providing these support strategies? If yes, please tell us about the challenges you encountered.	Open-ended
(7) Details on how you planned this support. Did you receive or consult any particular training or resources? If yes, where did you hear about these?	Open-ended
(8) Which training or resources did you consult or receive?	Open-ended
(9) To what extent was the information and training you received throughout your teacher training useful in supporting a child with JIA?	Open-ended
(10) How useful was the training or advice you received?	Open-ended
(11) Can you please tell us about any instances when, the training or information you received wasn’t of much use and you had to improvise on the training information or try something new of your own?	Open-ended
**Section C: Teaching Staff’s Support and Training Needs in Relation to JIA**	
Question	Type of question (and options of answers where relevant)
(1) On a scale of 0 (totally unconfident)*—*10 (very confident) how confident are you with supporting a child with JIA in school?	Closed 0 = totally unconfident 1 2 3 4 5 = neutral 6 7 8 9 10 = Very confident
(2) Which aspects of this support do you feel confident about? Please also tell us why you feel this way.	Open-ended
(3) Which aspects of this support do you not feel confident about? Please also tell us why you feel this way.	Open-ended
(4) To what extent did you feel supported by members of your Senior Leadership Team, School Governing Body and Key Stage Leaders in your role? Did you ever require assistance with making decisions, reporting concerns or ensuring inclusion for the child?	Open-ended
(5) Did you ever need assistance from another member of staff with carrying out your job role? If so, how did they support you?	Open-ended
(6) Were you the only individually trained staff member or was there a supportive member of staff also trained? If so, what skills did you use to ensure you worked successfully and supportively together?	Open-ended
(7) Is there anything that would be helpful in further improving your confidence in supporting a child with JIA in your classroom?	Open-ended
(8) According to you, what type of training or resources do you think would benefit teachers who are looking for advice on how to support a child with JIA in their class?	Open-ended
(9) Lastly, if you were to conduct a seminar or a training programme for teachers to care for children with JIA, what topics would you include?	Open-ended

## Appendix B. Semistructured Interview Schedule

This interview is about understanding and supporting primary-school-aged children with juvenile idiopathic arthritis (JIA). JIA has been found to affect a number of areas of young people’s lives, for example, their physical, social, and emotional wellbeing. In this interview, we would like to understand more about these impacts and specifically with respect to how they affect children’s learning and schooling experiences. We want to explore your views and understanding of working with young people who are affected by JIA.

The only people who will be able to hear this interview are the researchers on our project team. Your names and all identifying features of your school, community, and any cases you discuss will be anonymized on the written transcriptions of the interviews. The audio files will then be deleted and stored on our security-enabled university server. These files will be the only data that will be included in any written outputs from this study.

### Appendix B.1. Experiences of Supporting Children with JIA

(1a) What is your experience of supporting children with JIA in school?

*Prompts:* How many children with JIA have you supported in school? Over how many years have you worked with children with this condition? Which year were/are these children in?

(1b) Can you tell me about your role and responsibilities in relation to supporting children with JIA in school?

*Prompts:* Which strategies did you put in place to support the child/children with JIA in your school? Can you share how fruitful you believe these support strategies were? Where there any challenges in providing these support strategies?

(2) From your experience of supporting children with JIA, what is the nature of and presentation of the condition in school?

*Prompts:* is the condition stable or are there flare-ups? Are there periods of the week, term or academic year in which the symptoms worsen?

### Appendix B.2. Educational Impact of the Physical Effects of JIA

(3) Can you tell me a bit more about the physical effects of JIA for the children you have worked with?

(4) Can you tell me about how the physical effects of the condition affect children’s schooling experiences in relation to the following areas:

(a) Attendance?
(b) Curriculum subjects that involve physical activity (PE, drama, music, arts)?
(c) Core curriculum areas (numeracy, literacy, science)?
(d) Extra-curricular activities?
(e) School trips?

(5) What adaptations does the school make for the child in order to address these issues?

### Appendix B.3. Children’s Social Wellbeing in School

(6) Can you tell me about whether and how managing JIA can affect children’s social experiences in school? Such as for example:

(a) Children’s seating plans with peers in lessons?
(b) Seating arrangements for lunchtime?
(c) Participation in group work?

(7) How do children with JIA experience break and lunchtimes?

*Prompts:* Where do they go? Who are they with? What do they do during these times?

(8) Have the children with whom you have worked ever experienced exclusion or bullying on account of their condition? If yes, can you elaborate on what form this bullying took?

(9) Are there any social events or opportunities that children have been denied due to the nature of the condition?

*Prompts:* School events or field trips, residential opportunities, extra-curricular activities

(10a) To what extent do the children with JIA that you have worked with believe that their friends understand how their condition affects their school lives?

(10b) To what extent do the children with JIA that you have worked with believe that their teachers understand how their condition affects their school lives?

### Appendix B.4. Emotional Wellbeing in School

(11) Can you tell me about how JIA can affect children’s emotional wellbeing in school?

*Prompts:* Frustration, Sadness, Loss, Anger, What are the key triggers that provoke these feelings?

(12) What is the experience of pain that children with JIA suffer and how does this alter over the course of the school year?

*Prompts:* Periodic vs daily, in response to specific activities, better or worse at key seasons?

(13a) What are the strategies you put in place for pain management in a school environment?

*Prompts:* Are there any processes for administering pain management medication? What do you know about effective non-pharmacological pain management techniques?

(13b) How successful do you believe these pain management strategies were/are?

### Appendix B.5. Children’s Engagement in Lessons and Learning

(14) Can you tell me about how JIA can affect children’s learning and concentration in lessons?

*Prompts:* Periodic, related to specific curriculum areas?

(15) Are any of these issues affected by periods of assessment or testing in school?

(16) Can you tell me about how JIA can affect children’s completion of assessments?

Prompts: Marks?

(17) What adaptations does the school make for the child in order to address these issues with concentration and assessment completion?

(18) Is there anything we have not yet discussed that you feel would be helpful for us to know in relation to supporting a child with JIA in a school setting?

## Appendix C. Support Strategies Employed by Teaching Staff to Support Primary-School-Aged Children with JIA in a School Setting

**Category of Support Strategy**	**Specific Support Strategy or Characteristic of Support Strategy**
Strategies aiming to recognize and assess pain levels	Use of colored cards to notify teachers of pain
	Use of “traffic light colors” or “faces” to assess and indicate the level of pain
	Use of aids by children as an indication of pain state
	Proximity in class assisted monitoring
Adaptations and use of aids	Providing indoor space during breaks and lunchtime
	Assisting handwriting (pencil grips; laptop to type work; touch-typing programs; dictation/scribe; occupational therapy hand exercises; encourage breaks)
	Assisting sitting (provide a chair, bench, or a cushion)
Opportunities for rest & participation according to child’s capability	Regular breaks during activities and throughout school day
	Reduced school day
	Participation led by child’s capability
Qualities characterizing strategies	Discreet
	Socially inclusive
	Flexible
